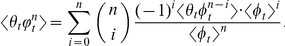# Stochastic Ontogenetic Allometry: The Statistical Dynamics of Relative Growth

**DOI:** 10.1371/journal.pone.0025267

**Published:** 2011-09-23

**Authors:** Anthony Papadopoulos

**Affiliations:** Department of Biological Sciences, Texas Tech University, Lubbock, Texas, United States of America; Genentech Inc., United States of America

## Abstract

**Background:**

In the absence of stochasticity, allometric growth throughout ontogeny is axiomatically described by the logarithm-transformed power-law model, 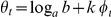, where 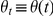 and 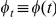 are the logarithmic sizes of two traits at any given time *t*. Realistically, however, stochasticity is an inherent property of ontogenetic allometry. Due to the inherent stochasticity in both 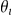 and 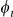, the ontogenetic allometry coefficients, 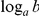 and *k*, can vary with *t* and have intricate temporal distributions that are governed by the central and mixed moments of the random ontogenetic growth functions, 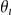 and 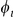. Unfortunately, there is no probabilistic model for analyzing these informative ontogenetic statistical moments.

**Methodology/Principal Findings:**

This study treats 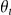 and 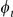 as correlated stochastic processes to formulate the exact probabilistic version of each of the ontogenetic allometry coefficients. In particular, the statistical dynamics of relative growth is addressed by analyzing the allometric growth factors that affect the temporal distribution of the probabilistic version of the relative growth rate, 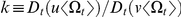, where 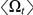 is the expected value of the ratio of stochastic 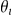 to stochastic 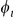, and 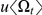 and 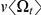 are the numerator and the denominator of 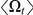, respectively. These allometric growth factors, which provide important insight into ontogenetic allometry but appear only when stochasticity is introduced, describe the central and mixed moments of 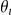 and 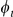 as differentiable real-valued functions of *t*.

**Conclusions/Significance:**

Failure to account for the inherent stochasticity in both 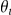 and 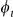 leads not only to the miscalculation of *k*, but also to the omission of all of the informative ontogenetic statistical moments that affect the size of traits and the timing and rate of development of traits. Furthermore, even though the stochastic process 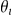 and the stochastic process 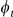 are linearly related, *k* can vary with *t*.

## Introduction

The most notable contributor to the mathematical analysis of allometry is J. S. Huxley, who in 1924 published a seminal paper in which he proposed that the power-law function (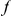) be used to describe allometric growth [1]:
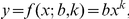
where *y* is the size of a trait, *x* is the size of another trait, and *b* and *k* are useful descriptors of allometric growth [1], [2]. Since then, numerous papers that support 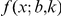 as a model for allometric growth have been published. One paper, in particular, shows that 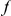 is an axiomatic functional form of allometry [3]. In theory, this suggests that the composite model 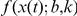, in which extrinsic time *t* is treated explicitly, yields an exact correspondence between 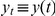 and 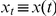, assuming that there is no stochasticity in 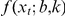 [4]:
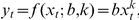
where 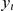 and 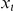 are the sizes of two ontogenetically related traits at any given *t* [4]. In reality, however, 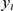 and 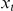 are inherently correlated stochastic processes, which are correlated random variables that depend on the deterministic variable *t*. It is not known with certainty the value of 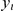 and the value of 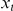 until after their measurements have taken place. Thus, 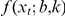 is exact, but unrealistic, only as a deterministic model. Subsequently, when the relationship between the realizations of stochastic 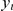 and the realizations of stochastic 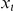 is described by 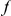, the probabilistic version of either *b* or *k* is not always constant with *t*. In fact, as this paper will show, the statistical moments of the random ontogenetic growth function for 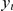 and for 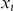 affect the temporal distribution of both *b* and *k*. This phenomenon has significant implications with regard to organismal form and function. And so the objectives of this study are to first incorporate stochasticity into 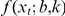 by treating 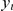 and 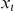 as correlated stochastic processes, thereby formulating an exact probabilistic model for allometric growth that applies throughout the ontogeny of any organism, and then to analyze the ontogenetic statistical moments that specifically govern the temporal distribution of *k*.

Unlike *b*, *k* is an important descriptor of relative growth [1], [5]–[7]. In ontogenetic studies of allometry, *k* is the coefficient of interest because it describes the specific growth rate of 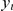 relative to the specific growth rate of 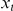 [1], [5]–[7]. Thus, the dimensionless ontogenetic allometry coefficient, *k*, is commonly referred to as the relative growth rate. Since allometric growth is inherently a stochastic process, *k* must be defined via stochastic analysis; but before this is done, it is necessary to first discuss important mathematical concepts, definitions, and notations used throughout this paper.

### Definitions and notations

Suppose 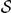 is a probability space on which the stochastic process 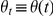 is defined. If 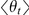 is the expected value (also known as the first statistical moment or the probability average) of 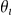, then the *n*th central moment of 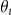 is 
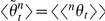
, where 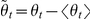, 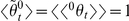 at every 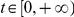, and 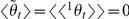 at every 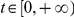. Now suppose 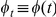 is another stochastic process defined on the probability space 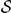. Then the probability covariance between 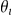 and 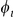 is 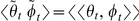; an obvious extension to this relation is the important identity 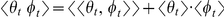. Thus, the *n*th mixed moment of 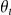 and 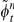 is 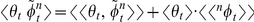. All of the stochastic processes involved in this study are defined implicitly as evolutionary, not stationary, random functions of *t*. With regard to the variable *t*, 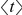 equals *t*, and 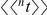 equals zero for every 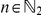. These equivalences hold for any deterministic process.

### Ratio of first-order deterministic *t*-derivatives

Let 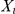 be the set of all deterministic or stochastic ratios of differentiable functions of *t*, and let 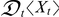 be the set of all ratios of first-order deterministic *t*-derivatives. Then, for any 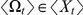, 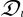 is defined by
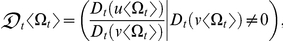
where 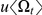 and 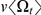 are the numerator and the denominator of 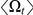, respectively. Therefore, 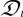 is a multivalued differential operator defined as the ratio of the standard first-order differential operator 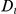:
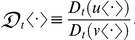
An important property of 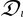 is that it operates linearly on sums of ratios of differentiable deterministic functions in which the denominators are common. For example, 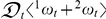 equals 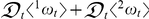 if 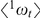 and 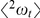 are expressed with a common denominator.

### The mathematical analysis of *k*

Let 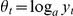 and 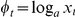 each be a deterministic ontogenetic growth function such that 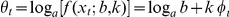 and 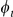 are deterministic variables that depend on *t*. Also, let 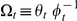 be the ratio of 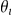 to 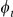. Then the first-order derivative of the deterministic ontogenetic growth function 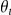 with respect to the deterministic ontogenetic growth function 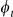 is [1], [5]–[7]

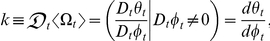
where 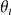 and 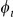 are differentiable real-valued functions of *t*. Note: 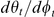 is a parametric derivative in which 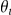 and 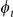 are differentiable deterministic functions. The temporal distribution of *k* has been a subject of intense interest (*see* [6] and [7]). The reason for this is that ontogenetic processes govern the size of traits and the timing and rate of development of traits [7]–[11]. Thus, *k* can vary with *t* [5]–[7]; this implies that the relationship between 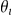 and 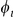 may not always be linear [5]–[7]. When 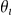 and 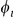 are linearly related, 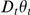 is proportional to 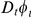 [1]; *k* is constant with *t*, and so the relationship between 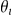 and 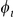 is described by 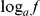. In contrast, when 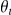 and 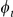 are nonlinearly related, 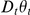 is not proportional to 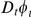 [5]; *k* varies with *t*, and so the relationship between 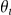 and 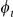 is not described by 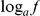. Both cases have been observed experimentally (*see* [12] and [13]). Although deterministic log-linear allometric growth trajectories are always the result of 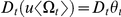 being proportional to 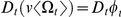, the proportionality between 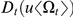 and 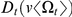 is not always expected to hold under stochastic log-linear allometric growth trajectories because 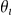 and 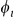 are correlated stochastic processes; their probability distributions interact in ways that are not intuitively obvious. The following is a case in point.

Since 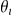 and 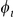 are inherently correlated stochastic processes, 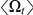 contains the central and mixed moments of those processes (Methods, equations 6–8). These statistical moments are described by the allometric growth factors (*see* Methods, equation 9) that affect the temporal distribution of 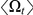. Of course, 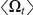 must be transformed into its probabilistic derivative, 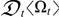, in order to analyze the allometric growth factors that affect the temporal distribution of *k*. These allometric growth factors, which only appear in the probabilistic version of 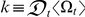, are essential because they provide important insight into ontogenetic allometry. Failure to account for the inherent stochasticity in 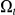 leads not only to the miscalculation of *k*, but also to the omission of all of the informative central and mixed moments of the random ontogenetic growth functions that govern the statistical dynamics of *k*. Therefore, by treating 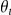 and 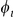 as correlated stochastic processes, this study reveals and analyzes the allometric growth factors that affect the temporal distribution of *k*.

The probabilistic derivative, 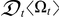, in which 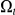 is a ratio of correlated stochastic processes, is newly presented in this study as the inner mean derivative of a random function with respect to a random function. This derivative implies the differentiation of the expected value of a random function with respect to the expected value of a random function, whereas the outer mean derivative of a random function with respect to a random function—for instance, 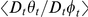
—implies the expected value of a ratio of correlated stochastic *t*-derivatives. In other words, 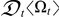, in which 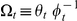 is a stochastic process, defines *k* as a deterministic variable, whereas 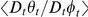, in which 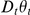 and 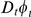 are stochastic, is the deterministic coefficient 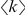. Although all of the statistical moments of *k* can be derived from 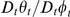, 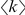 or 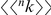 for any 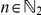 cannot vary with *t* because 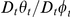 is simply a random variable, not a stochastic process. Thus, only 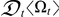, by which the deterministic variable *k* is defined, can vary with *t*. This distinction between the inner mean derivative 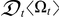 and the outer mean derivative 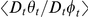 is important and is further addressed in the Discussion.

The concept of an inner mean derivative and an outer mean derivative only applies to the ratio of stochastic *t*-derivatives. The expected value of a stochastic *t*-derivative, such as 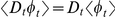, is simply referred to as a mean *t*-derivative (*see* equation 4.62 in [14]). Nelson [15] introduced mean derivatives (albeit based on the conditional expectation) to address issues in stochastic mechanics (*see* [16] and [17] for details).

The probabilistic version of 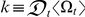 is not readily calculable because the numerator and the denominator of 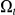 are correlated stochastic processes; the expected value of a ratio of correlated stochastic processes is generally not equal to the ratio of expected values of the stochastic processes [18]. Therefore, this study equates 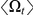 to its Taylor series expansion in order to reveal the central and mixed moments of the stochastic processes on which 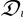 operates (Methods, equations 6–8). Although 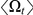 can be expanded as 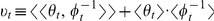 (which is not the Taylor series for 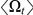), 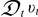, like its identity 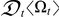, is not readily calculable because 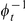 is stochastic. Subsequently, the Taylor series expansion of 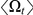 is essential for evaluating the probabilistic version of 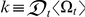. Also, 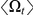 contains the term 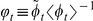, which is the ratio of 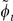 to 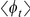 (Methods, equation 8). Naturally, 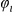 and 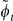 share similar statistical properties; for example, 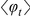 equals zero at every *t*, and 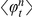 equals 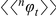 for every 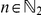.

### Results: The statistical dynamics of *k*

Using the definitions and notations described above, the inner mean derivative of the random ontogenetic growth function 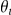 with respect to the random ontogenetic growth function 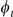 is (*see* Methods, equations 6–11, for derivation)
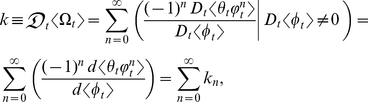
(1)where 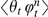 and 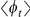 are differentiable real-valued functions of *t*. Equation (1) is the exact probabilistic version of *k*. This equation is also the exact general solution for the inner mean derivative of a random function with respect to a random function and can thus be applied to any ratio of correlated stochastic *t*-derivatives; no simplifying assumptions were made to derive equation (1). Note: 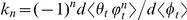 for each 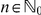 is a parametric derivative in which 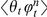 and 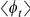 are differentiable deterministic functions.

Each of the *n*th terms in equation (1) is the statistical relative growth rate, 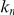, which can be expanded as (*see* Methods, equations 12 and 13, for derivation)
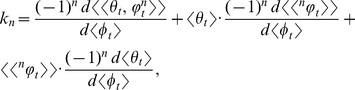
(2)where the summed terms
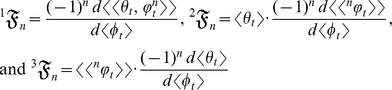
describe the allometric growth factors that affect the temporal distribution of *k*. The 0^th^ term in equation (1) is 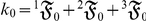 (where 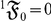 at every *t* and 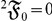 at every *t*), which becomes *k* either when 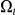 is deterministic or when 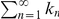 is zero at every *t*. Traditionally, 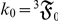 is calculated as *k* and is the ratio of 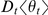 to 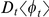 [19]. Note, however, that evaluating only 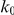 when 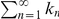 is not zero does not yield an exact *k* because the other terms—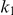, 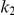,…,
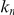
—must also be considered. Thus neglecting 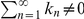 clearly leads to a miscalculated *k*. Moreover, *k* (or 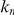 for every 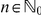) can vary with *t*; nonlinear allometries can occur, even though the stochastic process 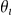 and the stochastic process 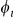 are linearly related.

The statistical dynamics of *k* can be readily analyzed by the summed terms (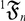, 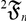, and 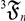) in equation (2). Consider the following example: let the stochastic processes, 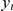 and 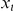, belong to the finite family of 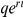
—the exponential growth-law functions in which only *r* is a random variable—such that the random ontogenetic growth function 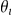 is 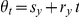 and the random ontogenetic growth function 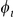 is 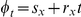. Then, if 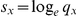 equals zero, equation (2) is (*see* Methods, equations 14–16, for derivation)
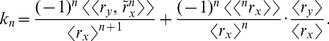
(3)The allometric growth factors in equation (3) are
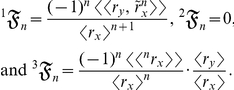
Equation (3) is an example of equation (2) in which the derivatives are explicitly defined. The appeal of this example (besides that it can be realistic for a particular organism) is that the allometric growth factors (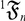 and 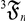) contain the slopes (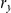 and 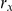) from 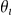 and 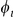, thus making it easy to interpret the biology of 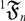 and 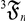. For instance, 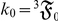 is simply 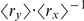; it is the ratio of 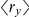 (the expected value of the specific growth rate of 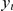) to 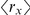 (the expected value of the specific growth rate of 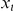). So, naturally, when the mean growth rate of 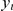 increases relative to the mean growth rate of 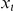, 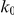 also increases. Note that *k* differs from 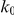 because 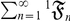 and 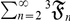 are nonzero sums. If 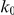 is 1 and 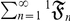 and 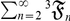 were both zero sums, then relative growth would be isometric [2]; however, since 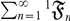 and 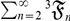 are really nonzero sums, relative growth deviates from isometry. This is a simple and yet realistic example illustrating the fact that *k* can be miscalculated if 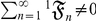 and 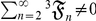 are not taken into account.

The statistical relative growth rate, 
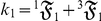
 (where 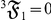), in equation (3) is
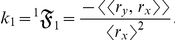
The nonzero coefficient, 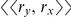, is the probability covariance between the random variable 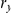 and the random variable 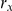; it is a measure of the joint distribution of 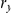 and 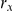. The more closely 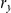 and 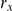 are positively associated, the lower the value of 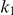 because 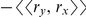 is less than zero. In contrast, the more closely 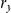 and 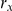 are negatively associated, the higher the value of 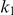 because 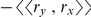 is greater than zero. And so whether 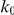 is being subtracted or added by 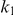 solely depends on the direction of association between 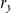 and 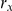.

The allometric growth factor (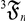) contains the term 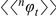, which in equation (3) is
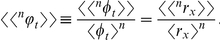
Clearly, 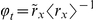 is a random variable, not a stochastic process. Thus, for instance, 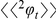 is a nonzero positive coefficient that represents the ratio of 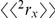 (the probability variance of 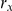) to 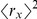 (the squared expected value of 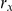). Consequently, 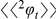 describes the ontogenetic variance of 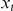, and 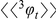 describes the ontogenetic asymmetry of 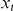. Both genetic and environmental factors can affect 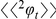 and 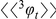, and these two ontogenetic statistical moments (or biological processes) influence *k* in a manner that is not intuitively obvious unless equation (1) is used.

It is important to note that the allometric growth factor, 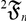, is zero in equation (3) only because 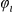 is a random variable, not a stochastic process; 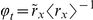 does not vary with *t* because 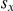 is constrained to zero, and thus 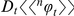 equals zero at every *t*. Since 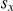 is constrained to zero, *k* does not vary with *t*.

Now suppose only 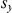 and 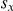 are random variables in the random ontogenetic growth functions, 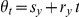 and 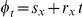. Then, if 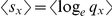 equals zero, equation (2) is (*see* Methods, equations 17–19, for derivation)
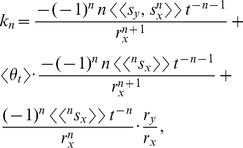
(4)where 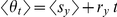 is a deterministic process and 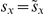 is a mean-centered random variable. In this case, 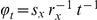 is a stochastic process because 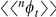 for each 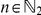 does not vary with *t*, and yet 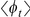 increases with *t* since there is growth. The allometric growth factors in equation (4) are
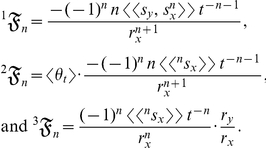
It is apparent that, unlike equation (3), equation (4) contains the deterministic variable *t*. Thus, *k* varies with *t*, and its values can either be greater than 1 (that is, positively allometric at every *t*) or less than 1 (that is, negatively allometric at every *t*) or an arrangement of both (that is, reversal in ontogenetic polarity) [10]. Note that 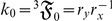 in equation (4) is constant with *t*; this implies that 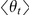 and 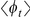 are linearly related, and so the relationship between 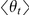 and 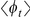 is described by 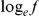. All other statistical relative growth rates (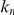 for every 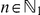), however, are derived from relationships that are not described by 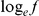 and therefore vary with *t*. For example, 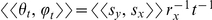 and 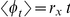 are nonlinearly related, and so 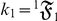, which is derived from the relationship between 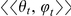 and 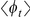, varies with *t*. Consequently, nonlinear allometries occur in this case, even though the stochastic process 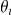 and the stochastic process 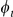 are linearly related.

Intricate temporal distributions of *k* can arise from the case described by equation (4). For example, suppose 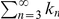 at every *t* is negligible compared to 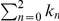 at every *t*. Then equation (1) is
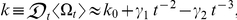
where 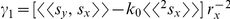 and 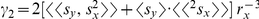 are probabilistic coefficients. Now there could be a condition in which the temporal distribution of *k* is not monotonic and is either positively allometric or negatively allometric: *k* has a stationary point at 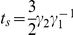 (set 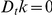 and solve for *t*), where the stationary value of *k* is 
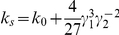
 (substitute 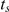 for *t* in *k*); thus, the temporal distribution of *k* is not monotonic. This is an interesting case because *k*, which could be either greater than 1 or less than 1 at every *t*, increases with *t*, reaches 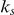 (the maximum rate of relative growth), and then decreases with *t*. This is classic case of accelerated and decelerated rates of relative growth within a given *t* period. Note that 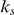 depends on the probabilistic coefficients, 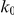, 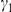, and 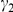. When 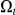 is deterministic, 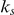 is undefined. Since, however, 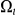 is inherently stochastic, the terms in 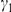 and in 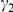 affect 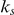 and 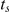. For instance, if 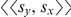 increases while 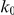, 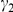, and all other terms in 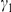 remain constant, then 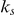 increases, assuming 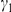 and 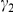 are positive. Moreover, increasing 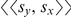 decreases the *t* at which 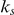 is reached; this is because 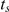 is inversely proportional to 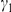, which is directly related to 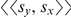. If stochasticity disappears, then 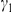 and 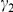 also vanish and 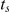 becomes undefined. So 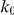, 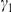, and 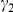 affect not only 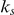, but also the *t* at which 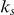 is reached. This is a clear case of how 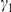 and 
—coefficients that only appear in the probabilistic version of 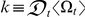
—affect the timing and rate of development of traits. Thus, ignoring the effects of stochasticity on both 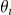 and 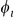 omits all of the informative ontogenetic statistical moments (e.g., 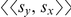) that govern the temporal distribution of *k*. Furthermore, even though the relationship between the realizations of stochastic 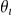 and the realizations of stochastic 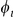 is described by 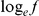, *k* differs from 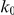 and can vary with *t*. This important fact should always be considered when analyzing allometric growth.

It is interesting to note that as *t* approaches infinity, equation (4) or any of its approximations reaches an asymptotic value of 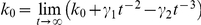. The *t* at which this asymptotic value is first reached is an indication of the cessation of the variability of *k* with respect to *t*. In other words, nonlinear allometries disappear as *t* approaches infinity. So as the allometric growth process evolves over *t*, two distinct phases are observed: the first phase is a non-uniform temporal distribution of *k*, and the second phase is a uniform temporal distribution of *k*. This two-phase allometric growth process may be more realistic than a growth process that exclusively corresponds to either the first or second phase. It should be made clear, though, that only the second phase is indicative to a log-linear allometric growth trajectory, since 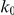 (not *k*) is constant with *t*. And so the probabilistic coefficients, 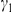 and 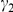, essentially have an insignificant impact on only the second phase of the allometric growth process. Clearly, the first phase of the allometric growth process can entail an intricate temporal distribution of *k*, such as the one provided in the previous paragraph.

Equations (3) and (4) are realistic examples of the types of temporal distributions of *k* that may arise from the random exponential growth-law function, 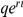, to which the stochastic processes, 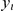 and 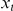, belong. The important distinction between equations (3) and (4) is the type of variable 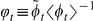 assumes: 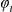 is a random variable (not a stochastic process) in equation (3); 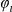 is a stochastic process in equation (4). As a result, *k* defined by equation (3) does not vary with *t*, whereas *k* defined by equation (4) varies with *t*. In either case, it is *q* or *r* that is a random variable. Nonetheless, it is entirely possible to have a case in which *q* and *r* are both random variables.

With regard to the convergence of equation (1), 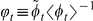 has an important role: equation (1) is guaranteed to converge at every *t* if the realizations of stochastic 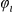 are between −1 and 1 at every *t*; this is because the realizations of 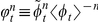 approach zero at every *t* as *n* approaches infinity.

## Discussion

 Although statistical models for relative growth have been developed (*see* [7] and [20]), their models, which show variability in 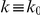 with respect to *t*, are not probabilistic because they do not incorporate actual stochasticity into 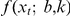; they do not treat 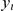 and 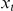 as correlated random functions. Also, although a probabilistic model for static (not ontogenetic) allometry, in which *x* is treated as an independent random variable (not as a stochastic process), has been proposed (*see* [21]), their model cannot address the statistical moments that govern the temporal distribution of *k* because their model is used to analyze the effects of stochasticity only on 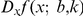. Consequently, equation (1) is entirely new and has no analog to any statistical model for relative growth previously developed.

Equation (1) is the exact general solution for the inner mean derivative of the random ontogenetic growth function 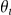 with respect to the random ontogenetic growth function 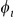. This equation, which is the exact probabilistic version of *k*, is general because it does not entail any simplifying assumptions. Thus, the generality of equation (1) makes it possible to analyze all of the informative ontogenetic statistical moments (or biological processes) that govern the temporal distribution of *k*:
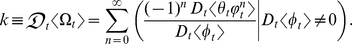



This expression makes it apparent that *k* is composed of an infinite series of ratios of first-order *t*-derivatives. The statistical complexity of *k* arises from the derivative in the numerator, which is the *t-*derivative of the *n*th mixed moment of 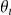 and 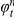. Each of these *n*th statistical moments is governed by the interactions between 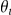 and 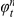. So most of the informative ontogenetic statistical moments are captured by the mean *t*-derivative, 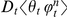; this is evident by expanding 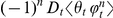 (*see* Methods, equations 12 and 13, for derivation):
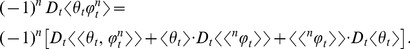
(5)


The summed terms in equation (5) compose the allometric growth factors (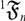, 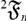, and 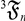) in equation (2). These allometric growth factors are important to interpret because they describe the central and mixed moments of the random ontogenetic growth functions that govern the statistical dynamics of *k*. Clearly, equation (5) is calculable, since each of the *n*th terms of 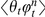 is a differentiable deterministic function of *t*.

To biologically interpret equation (5), one must specify the finite family of functions to which the stochastic processes, 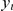 and 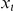, belong (*see*, for example, equations 3 and 4).

Equations (3) and (4) are examples of how to model and analyze the statistical dynamics of *k*. These examples are derived from the random exponential growth-law function that is theoretically assumed for a particular organism. Thus relaxing this assumption leads to the practical (experimental) side of modeling the statistical dynamics of *k*. Traditionally, 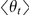 and 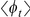 are experimentally measured, plotted with respect to each other, and then related by a differentiable function from which 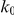 is derived [19]. This study, however, shows that 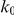 is not the only statistical relative growth rate that needs to be considered when evaluating *k* (*see* equations 1 and 2). The other statistical relative growth rates (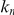 for every 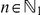) should also be quantified in a similar manner. For example, 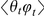 and 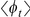 can be experimentally measured, plotted with respect to each other, and then related by a differentiable function from which 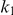 can be derived. Thus, the probabilistic version of 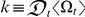 is a very practical metric: it only requires measuring the mixed and central moments of 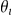 and 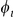.

The ontogenetic growth functions, 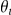 and 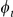, must be linearly related in order to satisfy the log-linear allometric function, 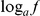. Thus, 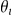 and 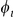 can be generalized as 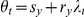 and 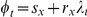, where deterministic or stochastic 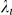 is any differentiable function of *t*. In equations (3) and (4), 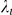 is simply *t*; but, to describe more intricate ontogenetic growth distributions, 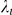 could also be 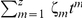 for any 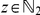, where 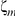 for each 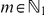 is a deterministic or stochastic parameter. Note that 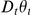 and 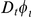 equals 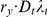 and 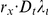, respectively; this is true for any distribution of 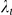. Subsequently, 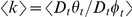 equals 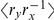, which is the expected value of the ratio of 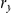 to 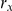.

 For most organisms, 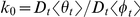 is constant with *t*; this implies that 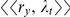 and 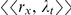 are typically zero at every *t*. In equations (3) and (4), where 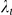 is *t*, 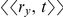 and 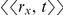 are naturally zero because *t* is naturally deterministic; thus, 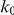 is naturally constant with *t* in these equations. There are some organisms (predominately plants) that show 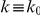 varying with *t* [22]. Indeed, this case, in which 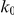 varies with *t*, is interesting to study, but complicates the biological analysis of 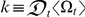 because the biological interpretation of 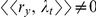 or 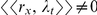 cannot explicitly be defined. Therefore, when analytically modeling 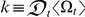, there is good reason to assume that 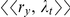 and 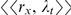 are zero at every *t*. Keep in mind, though, that while stochastic 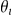 and stochastic 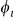 are linearly related, 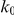 can vary with *t*.

It is important to note that if 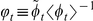 is not a stochastic process, then *k* (which differs from 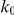) does not vary with *t* (*see* equation 3). If, however, 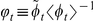 is a stochastic process, then *k* not only differs from 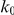, but also varies with *t* (*see* equation 4); this implies that the statistical relative growth rates (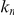 for every 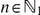) are derived from relationships that are not described by 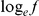, even though the stochastic process 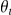 and the stochastic process 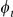 are linearly related.

Another important point to note is that 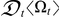 is mathematically different from the expected value of a ratio of correlated stochastic *t*-derivatives. If 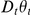 and 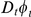 are correlated stochastic *t*-derivatives, then the outer mean derivative, 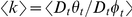, is generally not identical with equation (1). Stated more explicitly,
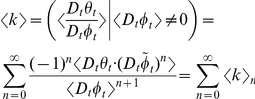
and
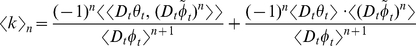
are generally not identical with equations (1) and (2), respectively. Note: 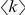 and 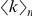 are derived in exactly the same manner as 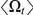 (*see* Methods, equation 8) and 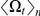 (*see* Methods, equation 9). Now compare the following limits: the outer mean derivative is
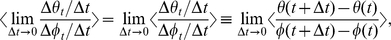
whereas the inner mean derivative is
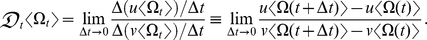
Thus, in 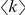, the limit operates on the ratio of stochastic 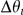 to stochastic 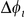; but in 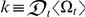, the limit operates on the ratio of deterministic 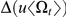 to deterministic 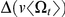. So 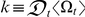 is identical with 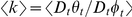 when both 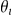 and 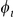 are deterministic or when only 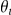 is stochastic. When, however, only 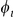 is stochastic or when both 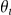 and 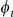 are stochastic, 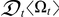 is generally not identical with 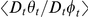 (*see* equation 4); the only exception is the special case when 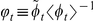 is not a stochastic process, but a random variable (*see* equation 3). As a result, the outer mean derivative 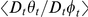 is a special case of the inner mean derivative 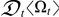. Also, 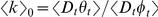 is equal to 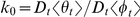.

In conclusion, equation (1) is completely versatile and has much to offer with regard to analyzing the allometric growth factors (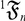, 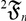, and 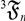) that affect the temporal distribution of *k*. When the derivatives in equation (2) are defined explicitly via specifying the random ontogenetic growth functions (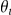 and 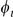), the allometric growth factors become biologically interpretable; they also become tractable in simulations, which are useful for modeling the statistical rates of relative growth for various distributions of 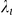 (*see* Methods, Simulating the probabilistic version of *k*). Thus, each of the statistical relative growth rates (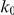, 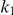,…, 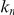), which are infinitely summed to form equation (1), can be analyzed in detail to reveal new insight into the statistical dynamics of relative growth.

Lastly, this study ignored the statistical dynamics of *b* because only *k* is an important descriptor of relative growth. But to obtain a complete characterization of the statistical dynamics of allometric growth, *b* or 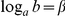 must also be considered. Since the stochastic analysis of *k* has been fully developed in this study (*see* Methods, equations 6–11), the exact probabilistic version of *β* can easily be formulated:
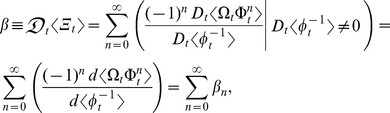
where 
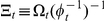
 is the ratio of 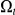 to 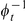 and 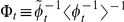 is the ratio of 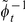 to 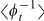. Each of the *n*th terms of 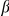 is the allometric growth descriptor, 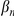:
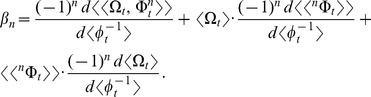
The summed terms in 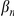 describe the allometric growth factors that affect the temporal distribution of *β*. The equation (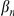) contains all of the ontogenetic statistical moments that govern the temporal distribution of *β*. And just like *k*, one could analyze the statistical dynamics of *β* simply by examining the summed terms in 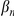. Note that, like 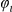 in equation (1), if 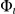 is a stochastic process, then *β* varies with *t*.

### Methods: The stochastic analysis of *k*

Let 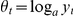 and 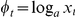 each be a random ontogenetic growth function such that 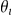 and 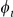 are correlated stochastic processes. Then, if 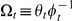 is the ratio of 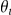 to 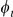, the expected value of 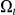 is
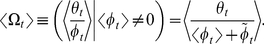
(6)Equation (6) contains the central and mixed moments of 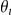 and 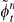. These statistical moments can be revealed by expanding equation (6) using the Taylor series generated by the function, 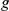, defined by the denominator 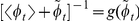 when α equals zero at every 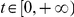:
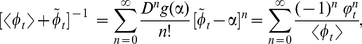
(7)where 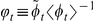 is the ratio of 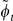 to 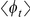. Substituting equation (7) into equation (6) yields
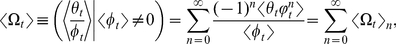
(8)where each of the *n*th terms in equation (8) is 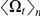:
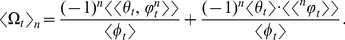
(9)The summed terms in equation (9) are the allometric growth factors that affect the temporal distribution of 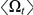. Rice and Papadopoulos [23] use a similar mathematical approach (that is, the Taylor series expansion of the expected value of the change in mean phenotype) to reveal important biological factors governing evolution.

Equation (8), which is the Taylor (or Maclaurin) series expansion of 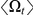, can also be expressed as 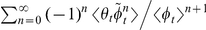. This particular expression, however, has no explicit common denominator, as its denominator has an unfixed exponent; thus, 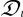 cannot operate linearly on this expression, and consequently fails to define *k* from this expression. In contrast, equation (1), in which 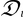 operates specifically on equation (8), uniquely defines the probabilistic version of *k*. Equation (8) is thus essential for evaluating 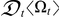: the *t*-derivative of the numerator in equation (8) is
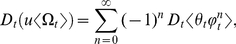
(10)and the *t*-derivative of the denominator in equation (8) is
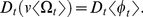
(11)Therefore, equation (1) (that is, the inner mean derivative of the random ontogenetic growth function 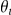 with respect to the random ontogenetic growth function 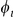) is the ratio of equation (10) to equation (11):
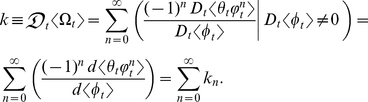
Now the identity 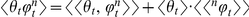 can be used to expand 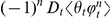:
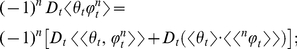
(12)the product rule is used to expand 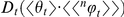:
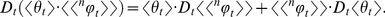
(13)Substituting equation (13) into equation (12) and dividing by 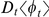 yields the expanded form of the statistical relative growth rate, 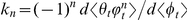:
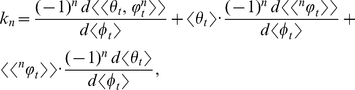
which is identical with equation (2). The summed terms in equation (2) are the allometric growth factors (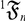, 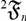, and 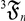) that affect the temporal distribution of *k*:
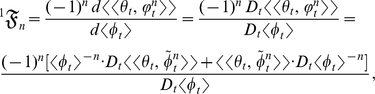


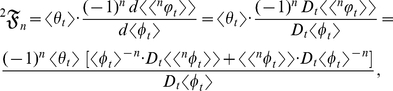
and
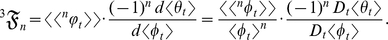
When 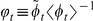 is a stochastic process, the product or quotient rule can be used in 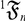 and in 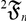 to calculate their derivatives. Note that 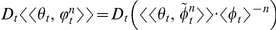 and 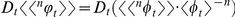 represent deterministic *t*-derivatives of the product of two deterministic functions.

Now suppose for a particular organism the random ontogenetic growth functions, 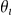 and 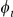, are defined by 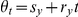 and 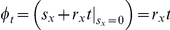 in which only 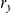 and 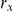 are random variables. Then the allometric growth factors, which are the summed terms in equation (2), are as follows:
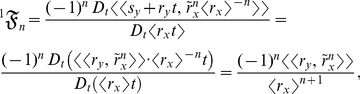
(14)

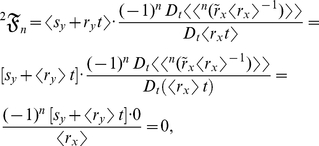
(15)and
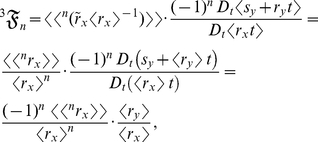
(16)where 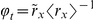 is a random variable, not a stochastic process. Summing equations (14), (15), and (16) then yields equation (3):
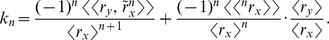
If, however, 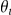 and 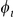 are defined by 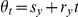 and 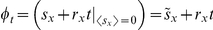 in which only 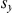 and 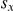 are random variables, then the allometric growth factors are
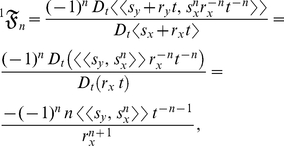
(17)

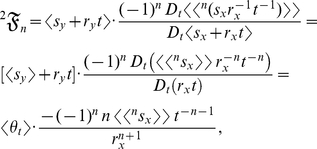
(18)and
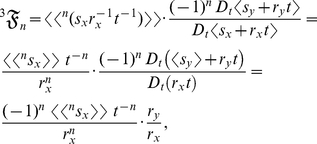
(19)where 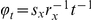 is a stochastic process and 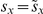 is a mean-centered random variable. Summing equations (17), (18), and (19) then yields equation (4):
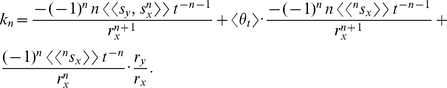



### Methods: Simulating the probabilistic version of *k*

Simulating 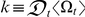 using 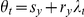 and 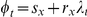 as correlated random functions can easily be done: first specify the terms in 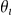 and in 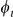 that are stochastic and then provide their (joint) probability distributions. Because the stochastic process 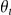 and the stochastic process 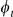 are linearly related and because 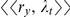 and 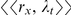 are assumed to be zero at every *t*, 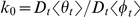 is constant with *t*. Thus, the parametric derivative, 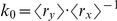, is readily calculable, since 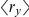 and 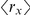 are known from the distribution of 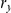 and the distribution of 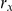, respectively. In contrast, 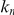 for each 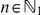 is not readily calculable, but can easily be assessed in simulations by first evaluating 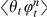 for each 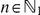 and then relating 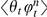 to 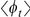 by a differentiable function from which the derivative (i.e., 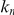) can be calculated. So, for example, 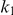 is the parametric derivative, 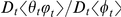; to evaluate 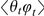 properly in simulations, the following identity of 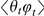 should be used: 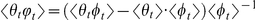; this is because 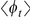 is evaluated together with (not separate from) 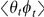 and 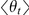 in simulations. Therefore, the binomial expansion of 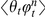 is useful for numerically evaluating 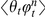: